# Specialism in Medicine

**Published:** 1881-09

**Authors:** 


### SPECIALISM IN MEDICINE.

Dr. A. Reeves Jackson, of Chicago, in a paper read before the Illinois State Medical Society, related to what extent this had existed in ancient Egypt, and said that history had repeated itself in our age. The chief causes for such a division of labor in medicine were an increase in professorships, a lack of medical knowledge in the general practitioner, who could not make a practical study of his art in the colleges; or, if a few did, "it was altogether accessory and necessitated an accessory fee." The consciousness of possessing little experience in many cases would lead the young physician to call a specialist in consultation ; it was the only proper course, although humiliating. Many found it impossible to avail themselves of all the necessary knowledge, and studied only one subject, while others trusted to luck and did not study at all, well knowing that "nine cases out of ten will get well any way." The college faculties having become aware of this necessity for longer training, some attempted to remedy it "for a consideration." A post-graduate course had thus been established. It was a "repetition of the old story," consisting in lectures and demonstrations, without experimental practice. Precept without example was insufficient, and the various branches taught were only suggestive of a useful treatment.

Specialists were necessary to meet the demand for special skill, and was it not for this the progress of medicine had been arrested. Great men were all specialists, and concentrated energy was the most powerful. Among its evil results, however, were conceit, a proneness to magnify slight ailments, a tendency to originate "men of one idea," and sometimes it would lead to errors in diagnosis. But these were not at all direct results. Men unacquainted with general medicine, or students just out of school, had entered specialism, which here ceased to be specialism to become quackery, sometimes professional quackery. But as no organ carried on the process of life without the participation of all other organs, it was necessary to become well acquainted with all before treating one in particular. Specialists should be welcomed by the profession, as it is the outgrowth of the latter; but they must first be intelligent general practitioners.—*Medical Record.*

